# Experimental investigation and simulation analysis of cast-steel joints under vertical pressure

**DOI:** 10.1038/s41598-024-62138-4

**Published:** 2024-05-17

**Authors:** Zhihao Li, Yizhong Zhang, Wenfeng Du, Liming Zhu

**Affiliations:** School of Civil and Architectural Engineering, Henan University, Kaifeng, Henan China

**Keywords:** Cast-steel joint, Tree-like column structures, Full-scale model test, Engineering, Civil engineering

## Abstract

The joint made of cast steel is frequently utilized within a treelike column structure to ensure a smooth transition. It is of great significance in ensuring the overall structural safety, but currently, the mechanical property and bearing capacity of this type of joint cannot be fully understood. This study investigates the load characteristics of three-forked cast steel joints through concrete experiments, finite element analysis, and regression method formula derivation, filling the gap in mechanical properties and calculation formulas of forked cast steel joints. Initially, a comprehensive model of the cast-steel joint, sourced from a practical engineering, underwent vertical load testing. Detailed scrutiny of stress distribution and vertical displacement of the tested joint was conducted based on the experimental outcomes. Subsequently, a finite element model of the tested joint was constructed using SolidWorks and subjected to analysis via ANSYS. The numerical findings were juxtaposed with experimental data and extrapolated to encompass other parametric scenarios. Ultimately, a regression analysis method was employed to derive a calculation formula for the load-carrying capacity of branch-bearing cast-steel joints. The regression analysis method can accurately obtain the load-bearing capacity calculation formula for tree-shaped joint models and can be extended to determine corresponding branch and main pipe dimensions, as well as the deviation angle between branches and the main pipe, under known load conditions. This improves design efficiency and accuracy. Comparative analysis reveals a substantial concurrence among experimental, finite element analysis, and formula-based predictive outcomes. The maximum error between experimental results and those obtained from finite element analysis is 9.02%. The maximum error between the results calculated using the load-bearing capacity formula derived from regression methods and those from finite element analysis is only 1.9%.

## Introduction

The tree-like column has been widely utilized in engineering owing to its visually appealing structure and remarkable mechanical characteristics^1^. Since its introduction at Stuttgart Airport in Germany in 1991, several significant undertakings, such as Stansted Airport in London, the ION Orchard Center in Singapore, Detroit Airport in the United States, and the Railway Station in Changsha, China, have adopted this pioneering architectural structure^2–5^.

It is obvious that the joints connecting the backbone and branches play a crucial role in the treelike structure. Firstly, the entire upper framework relies solely on a singular connection, and the arboreal assembly risks collapse in the event of this connection's demise. Secondly, numerous elements, encompassing both the main stem and lateral tubes, converge at the connection with seamless shifts, rendering the mechanical properties of such connection intricate. Arslan et al.^3^ explored the developmental history of tree-shaped joints and analyzed various types of such joints, concluding that tree-shaped joints exhibit novel shapes and favorable load-bearing characteristics. Shadhan et al. conducted static load^6^ experiments on tree-shaped joints as well as axial and lateral combined load experiments^7^, investigating the favorable load characteristics of tree-shaped joints.

Tree-like column structures commonly employ cast-steel joints with branches^8^. In contrast to welded tubular joints, the use of cast-steel joints eliminates the residual stress caused by intricate welds at intersections. This not only simplifies construction but also accelerates the building process of tree-like structures^9,10^.

While the utilization of cast steel in structural applications has garnered consistent interest over recent decades, research regarding cast-steel joints remains in its nascent phase^11^. Conducting full-scale experiments serves as a direct and efficient means to comprehend the performance of cast-steel joints. Consequently, several experiments assessing cast-steel joints in significant large-span steel roof structures have been conducted, including those at Shanghai South Railway Station^12^, Beijing National Stadium^13^, and Chongqing Olympic Stadium in China^14^. Additionally, numerical analysis has emerged as another viable approach to delineate the stress distribution within cast-steel joints comprehensively. Finite element analysis results offer invaluable insights for cast-steel joint design, as evidenced by projects such as the Cycling Gymnasium for Beijing Olympic Games^15^, Guangzhou New Railway Station^16^, and Tianjin Convention and Exhibition Center^17^. Eurocode 3 presents the component method, facilitating the evaluation of joint stiffness and resistance characteristics by aggregating those of all constituent components^18,19^.

The existing building standards and specifications have not provided specific guidance for the design and analysis of cast steel bifurcation joints. The absence of this regulatory framework leaves designers and engineers at a loss in ensuring the safety and efficiency of these critical structural elements. In addition, research on the mechanical properties, calculation methods, and structural optimization of cast steel bifurcation joints is also relatively limited. Although traditional connection design and analysis techniques may provide some insights, they often cannot cope with the unique geometric configurations and load conditions of forked tree structures.

In response to these urgent needs, this study aims to comprehensively explore the mechanical behavior and application of cast steel bifurcation joints in bifurcation tree structures. Through a typical joint configuration consisting of one main trunk and three branches, combined with full-scale testing, finite element analysis, and mathematical formula derivation, an in-depth study was conducted on the bearing capacity of bifurcated tree shaped cast steel joints. Firstly, full-scale ultimate bearing capacity tests were conducted on the three branch tree joint, and then it was simulated using ANSYS version 2022R1 (64-bit) software. The stress results of the two were basically consistent, indicating that finite element analysis can effectively replace full-scale tests to a certain extent. Later, finite element analysis was used to analyze 24 different shape parameters of trident tree shaped joints, and the factors affecting the bearing capacity of the joints were obtained. Regression analysis was conducted on these factors, and a formula for deriving the bearing capacity of trident tree shaped cast steel joints was established based on this.

This study further verified the consistency between full-scale testing and finite element analysis of bifurcated cast steel joints, and filled the gaps in mechanical performance parameters and calculation formulas. It provides experimental and theoretical basis for future research on bifurcated tree joints, and the proposed bearing capacity derivation formula also improves the efficiency and accuracy of joint design. In addition, our research findings are expected to provide reference for the development of future building standards and regulations, thereby promoting the wider application of branched tree structures in modern architecture.

## Model test of the three-branch cast-steel joint

### Engineering background and design philosophy

**Figure 1:**
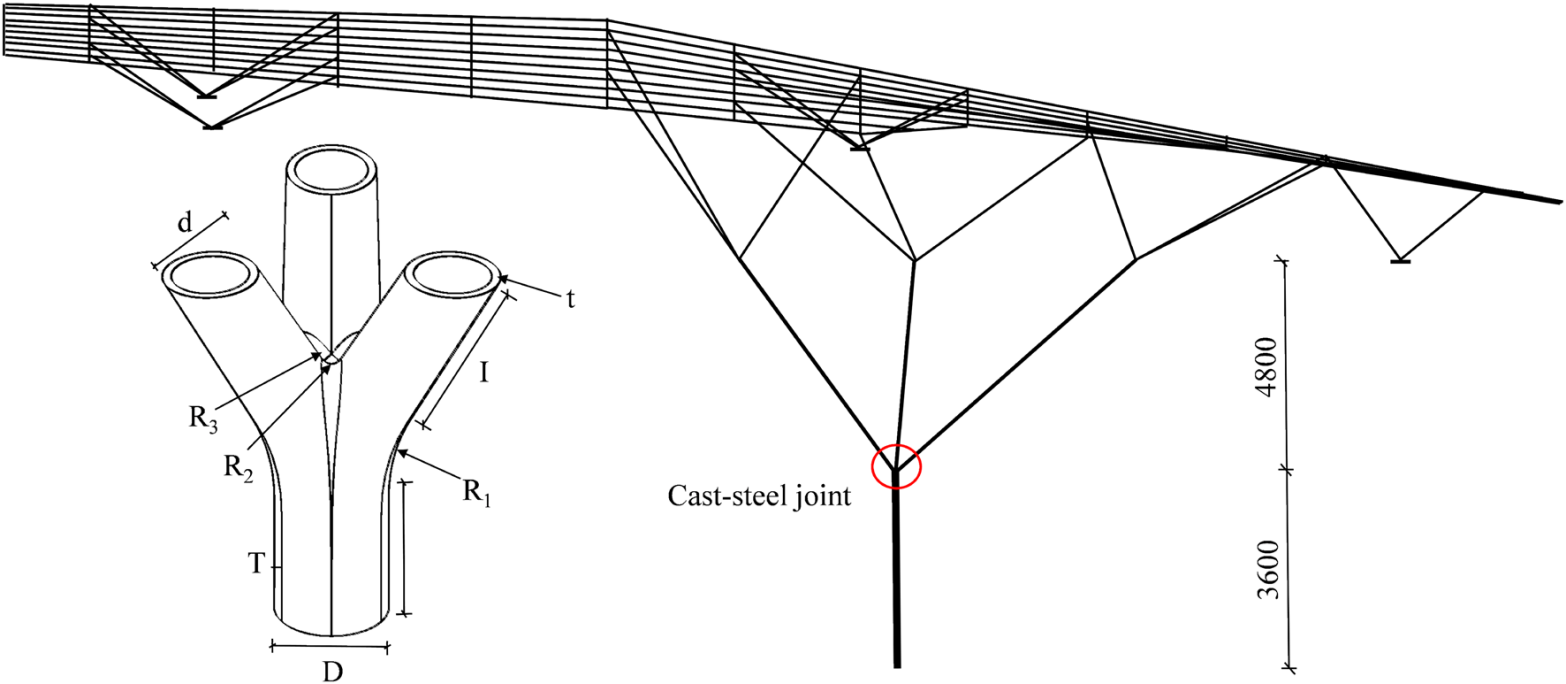
Structural and joint model for the exhibition hall of Zhonghong Hotel.

The design specifications for the exhibition hall at Zhonghong Hotel in Kaifeng City, China, outline dimensions of 24 m × 34 m. Illustrated in Fig. 1, the roof structure employs a single-layer latticed shell, supported by a tree-like column and four additional supports from reinforced concrete frames. Notably, a cast-steel joint was employed at the intersection point of the trunk and the first-level branches.

The decision to utilize a cast-steel joint was driven by three primary considerations. Firstly, the joint needed to be fabricated from thin-walled pipes to minimize weight. Given the complexity of achieving hollow joints with intricate shapes through conventional methods, casting was deemed the most viable option. The thickness of the joint matches that of the main pipe, set at 1/20 of the pipe diameter to meet production and load-bearing requirements. Secondly, to ensure smooth stress transmission across different parts of the joint, the lengths of both the main pipe and branch pipes within the joint zone were carefully designed to avoid being excessively short. Thirdly, for ease of production and installation, the branch pipes were horizontally cut flat. To fulfill these criteria, the cast-steel joint was meticulously designed, as depicted in Fig. 1.

The joint configuration comprises one main pipe and three symmetric branch pipes. The main pipe has dimensions of length (*L*) = 800 mm, diameter (*D*) = 500 mm, and wall thickness (*T*) = 40 mm. Each branch pipe shares identical dimensions: length (*l*) = 500 mm, diameter (*d*) = 350 mm, and wall thickness (*t*) = 35 mm. The chamfer radius (*R*_1_) between the main pipe and the branch pipes is 1000 mm, while the outside chamfer radius (*R*_2_) between branch pipes is 50 mm. Additionally, the inside chamfer radius (*R*_3_) among branch pipes is 20 mm. Other essential parameters include *θ* = 30° (angle between main pipe and branch pipe), *β* = 0.7 (ratio of outer diameter of branch pipe to outer diameter of main pipe), and *γ* = 20 (ratio of outer diameter of main pipe to main pipe wall thickness).

### Manufacturing and material properties of the joint specimen

The joint is fabricated from ZG20SiMn cast steel^20^, characterized by the following chemical composition: Carbon (C)—0.18%, Silicon (Si)—0.60%, Manganese (Mn)—1.50%, Phosphorus (P)—0.020%, Sulfur (S)—0.015%, Chromium (Cr)—0.30%, Molybdenum (Mo)—0.15%, and Nickel (Ni)—0.40%. ZG20SiMn steel has strong strength and stiffness, which can ensure the structural integrity and stability of joints. And corrosion resistance, wear resistance, and impact resistance, extending the service life of joints and reducing maintenance costs. And it has good fluidity and filling ability during the casting process, which can achieve complex shape casting.

**Figure 2:**
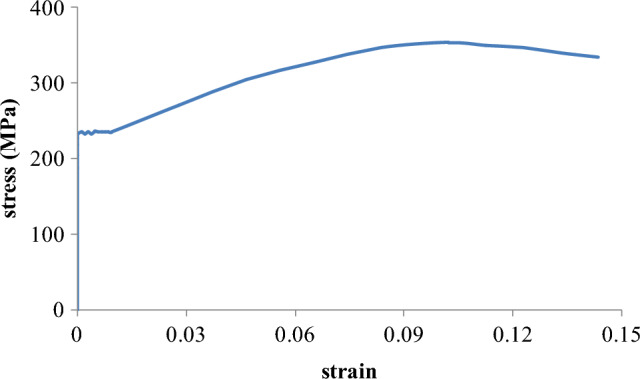
Stress–strain relationship of the cast-steel material.

The joint specimen was cast using a sand mold at Tengfei Factory in Xinxiang City, China. Following heat treatment and polishing, the specimen was transported to the structural laboratory of Henan University for testing. A standard dog-bone flat specimen was also prepared to evaluate the material properties^21^. The material test results, including the typical stress–strain relationship, are illustrated in Fig. 2. The stress–strain curve for the cast-steel coupon exhibits a distinct yielding plateau, indicating favorable material properties. The yield strength is measured at 235.7 MPa, and the ultimate strength reaches 353.6 MPa. The percentage elongation at fracture is found to be 25.9%, and the ultimate-to-yield strength ratio is calculated at 1.50. These results demonstrate that ZG20SiMn cast steel possesses notable ductility and strain hardening ability, exceeding the basic requirements stipulated in building codes such as Eurocode 3^22^. Specifically, the percentage elongation at fracture surpasses the minimum of 15%, and the ultimate-to-yield strength ratio exceeds the required minimum of 1.1.

To ensure precise adherence to design specifications, the size of the actual joint was meticulously measured, and the relative errors between the actual and design siz-es are detailed in Table 1. Notably, the maximum relative error recorded is 2.80%, comfortably below the acceptable threshold of 5.00%. This demonstrates that the pro-duction of the joint specimen successfully meets the stringent design requirements.

**Table 1:** The comparative analysis of the test-specimen dimensions.

Parts of the joint	Measured sizes (mm)	Design sizes (mm)	Errors (%)
The main pipe length (*L*)	806	800	0.75
The main pipe diameter (*D*)	495	500	0.10
The main pipe wall thickness (*T*)	39.5	40	1.25
The branch pipes length (*l*)	492	500	0.16
The branch pipes diameter (*d*)	345	350	1.71
The branch pipes wall thickness (*t*)	34.6	35	1.14
The chamfer (*R*_1_)	994	1000	0.60
The chamfer (*R*_2_)	20.46	20	2.30
The chamfer (*R*_3_)	51.4	50	2.80

To further reduce experimental errors, we have employed the following methods:

Calibration of measurement instruments: Prior to the experiment, all measurement instruments, including load cells, strain gauges, and data acquisition systems, underwent thorough calibration procedures. Calibration involved comparing the readings of the instruments against known reference standards to verify their accuracy and reliability.

Load application procedures: The procedures for load application were carefully designed to minimize errors and ensure uniform loading conditions. Load cells were calibrated and verified to ensure accurate measurement of the applied loads. The loading system was equipped with safeguards to prevent overloading and ensure controlled loading rates, minimizing the risk of sudden fluctuations in load that could affect the integrity of the test specimen.

Material testing: Material testing procedures were conducted to characterize the mechanical properties of the ZG20SiMn cast steel used in the experiment. Standardized test specimens were prepared and subjected to tension, compression, and bending tests to determine parameters such as yield strength, ultimate strength, and modulus of elasticity. These tests were performed in accordance with established testing protocols and standards, ensuring the accuracy and reliability of the material property data.

Data collection and analysis: During the experiment, data collection procedures were meticulously executed to ensure accurate and reliable measurements. Strain gauges were strategically placed at critical locations on the test specimen, guided by finite element analysis to capture variations in strain distribution. Data acquisition systems continuously recorded strain readings at predefined intervals, synchronized with load measurements to correlate strain with applied load.

### Loading procedure

**Figure 3:**
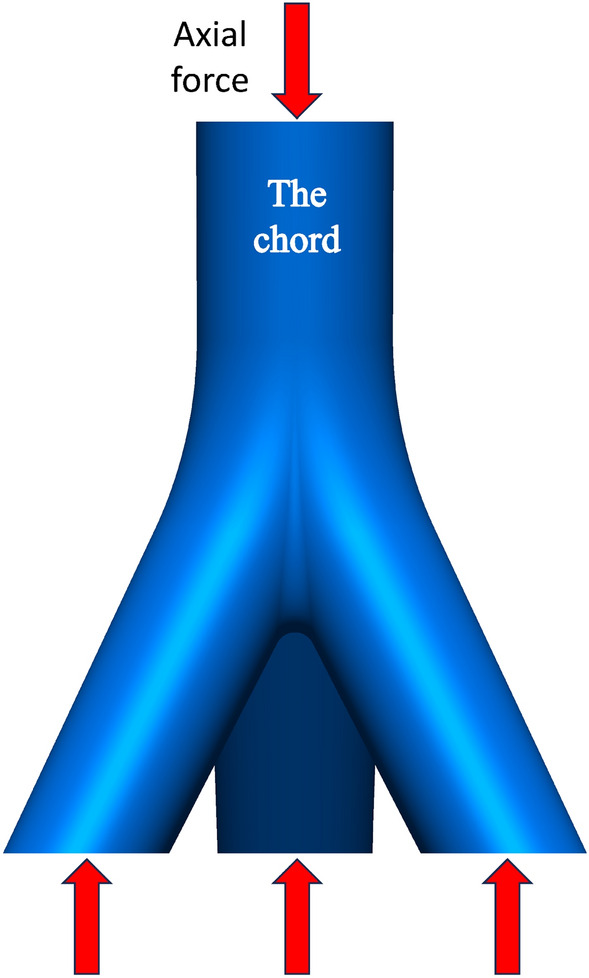
Force diagram of the joint.

The experimental setup involved applying a vertical load, as depicted in Fig. 3, with a maximum load capacity set at 5000 kN. To facilitate contact between the experimental joint and the load piston, an initial preloading stage was implemented, determining a preloading value of approximately 30 kN through several trials. Following preloading, the regular loading phase unfolded in five stages, each incrementing by 1000 kN at a constant rate of 10 kN/s. At the conclusion of each stage, the load was maintained for a duration of 30 s. Subsequently, the unloading process commenced, mirroring the loading stages with five steps, each decreasing by 1000 kN at a rate of 10 kN/s. Similar to the loading stages, a 30-s holding period followed the completion of each unloading stage.

### Strain measurement

Resistance strain gauges were employed to measure the strain in the cast-steel joint during testing. These strain gauges and strain rosettes were manufactured by the Giant Star Electric Measuring Element Factory in Taizhou, China. The specifications for the strain gauges are as follows: a resistance value of 120 ± 0.2 (Ω), a sensitivity factor of 2.08 ± 1%, and a sensitive grid size of 2 mm (gate length) × 1 mm (width) on a substrate measuring 4.5 mm (gate length) × 2.4 mm (width). Similarly, the strain rosettes possess a resistance value of 120 ± 0.3 (Ω), a sensitivity factor of 2.08 ± 1%, and a sensitive grid size of 3 mm (gate length) × 2 mm (width) on a substrate measuring 11.5 mm (gate length) × 11.5 mm (width).

**Figure 4:**
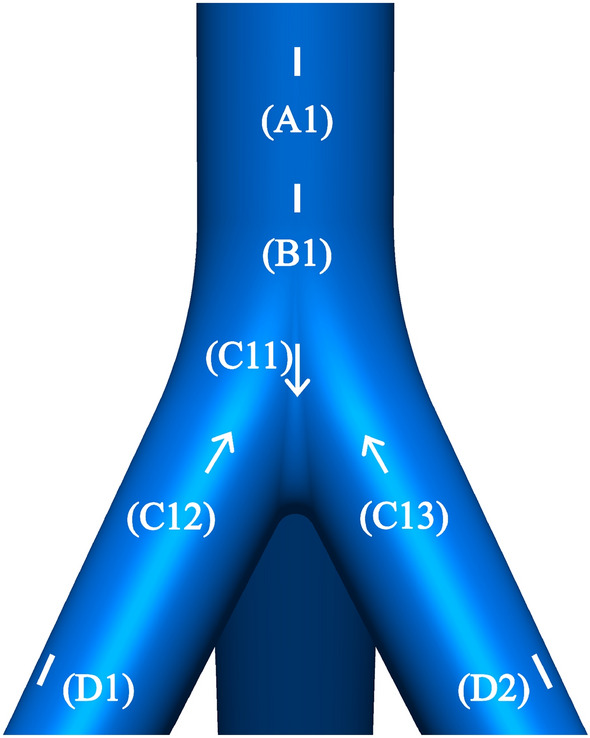
Layout of measuring points.

The surfaces of the joint were pre-polished to facilitate the attachment of strain gauges and strain rosettes. The placement of measuring points was primarily determined by the characteristics of joint stress calculated through finite element analysis. Analysis results indicated elevated stress levels near the joint core area, contrasting with lower stresses in the main pipe and branch pipes. Consequently, measuring points near the joint core area were densely positioned. Overall, measuring points were concentrated in four areas: (A) upper part of the main pipe, (B) lower part of the main pipe, (C) vicinity of the joint core area, and (D) branch pipes. Strain gauges were affixed to the upper and lower parts of the main pipe, where stress distribution is simpler. Conversely, strain rosettes were placed near the joint core area, characterized by unknown principal stress directions and complex stress distribution patterns. Measuring point positions were uniformly distributed and symmetrical, with three copies along the joint circumference. Figure 4 illustrates the facade of measuring points on the joint, with identical placement on the other two symmetrical sides. The specific locations and quantities of measuring points are outlined in Table 2.

**Table 2:** The stresses of the measuring points (MPa).

No.	1000 kN	2000 kN	3000 kN	4000 kN	5000 kN	Locations
A1	26.7	53.6	80.4	112.5	130.4	The upper part of the main pipe
A2	23.2	51.3	78.6	109.3	126.3	
A3	28.3	54.7	84.3	110.7	121.4	
B1	34.5	72.8	112.3	145.7	183.3	The lower part of the main pipe
B2	31.6	69.3	114.2	148.6	178.7	
B3	38.7	75.4	115.3	147.3	176.9	
C11	42.7	88.2	128.3	183.4	235.4	The vicinity of the joint core area
C12	44.3	87.6	130.1	179.5	235.3	
C13	44.7	87.4	126.8	182.4	235.7	
C21	44.8	85.7	127.2	178.6	235.2	
C22	45.3	86.3	128.4	185.1	235.3	
C23	41.9	86.7	129.1	179.3	235.6	
C31	42.4	89.2	128.6	180.1	235.4	
C32	46.3	84.9	131.2	185.3	235.3	
C33	44.7	85.4	127.9	176.1	235.1	
D1	35.2	71.7	110.6	144.3	181.4	The branch pipes
D2	34.6	70.8	117.3	151.5	179.4	
D3	32.7	68.7	108.4	156.3	183.5	

### Experiment observation

**Figure 5:**
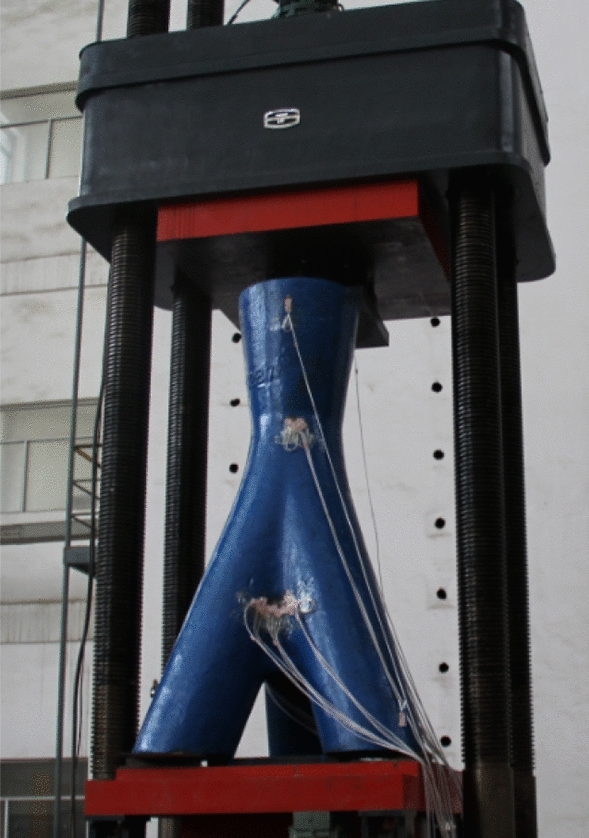
Test-specimen and loading device for the experiment.

At the onset of preloading, a faint sound was discernible, attributed to the adjustment of the experimental specimen to close the gap between the piston and itself. This sound dissipated as the load reached 20 kN. Following the completion of preloading, the regular loading test proceeded in accordance with the prescribed loading procedure. Figure 5 illustrates a photograph capturing the joint under load. Throughout the loading process, both the measured displacement and strain exhibited consistent, uninterrupted development without any notable jumps.

### Analysis and discussion of test results

#### Analysis of the load–displacement curve

**Figure 6:**
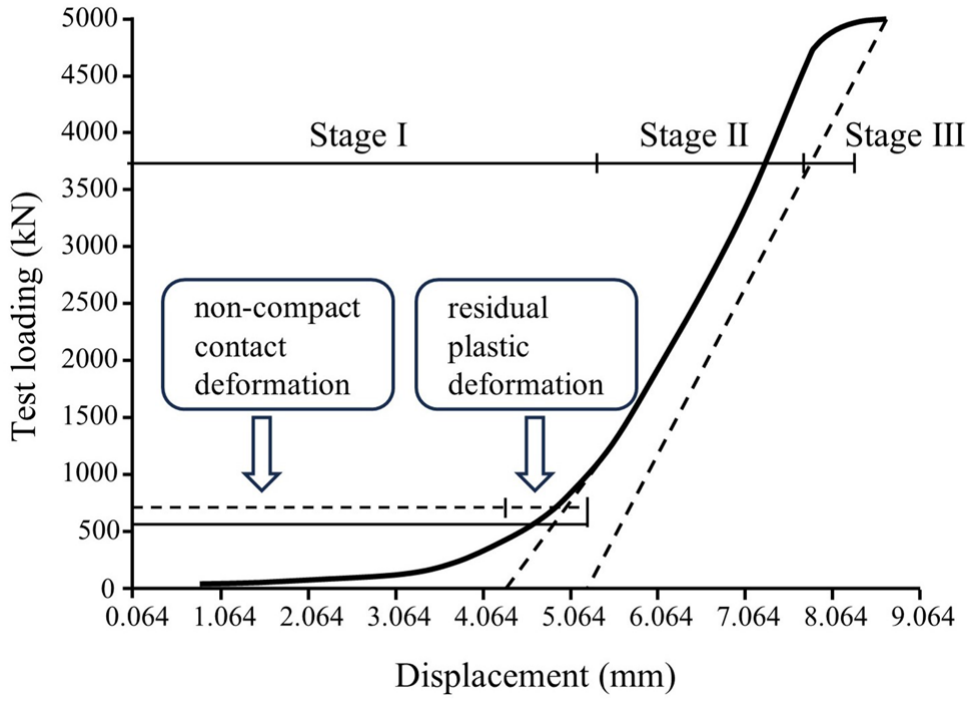
Load-deformation curves of specimens.

Figure 6 depicts the load–displacement curve obtained from the experimental specimen. Broadly, the load–displacement curve of the joint can be delineated into three distinct stages. In stage I, characterized by relatively low loads (less than 500 kN), the slope of the load–displacement curve is minimal. This signifies a significant increase in joint displacement at the initial stage of loading, attributable to the non-tight contact between the joint and the testing equipment.

With the continuous increase in load, the load becomes proportional to displacement, but the slope of the load–displacement curve experiences a noticeable augmentation. This phase is defined as stage II, suggesting that the joint operates within the elastic state as the load varies between 500 and 4750 kN.

As the load approaches the maximum design value (stage III), the load–displacement curve exhibits a non-linear behavior, indicating that the joint transitions into the elastic–plastic state. Notably, a significant increase in displacement corresponds to a minor increment in load. The slope of the load–displacement curve in stage III indicates a rapid expansion of the plastic zone within the joint. Therefore, it is advisable to consider the proportional limit observed in stage II as the control value for the design of the cast-steel joint with branches. From stage II, it is evident that the ultimate bearing capacity of the joint specimen is 4750 kN.

During the unloading process, the joint's deformation exhibited linear recovery in tandem with the decrease in load. The maximum vertical displacement recorded throughout the entire process amounted to 8.305 mm. However, upon unloading, the joint did not revert to the zero point due to residual deformation. This residual deformation can be attributed to two factors. Firstly, non-tight contact between the joint and the testing equipment, estimated to be approximately 4.435 mm (refer to Fig. 6). Secondly, residual plastic deformation, which amounts to about 0.890 mm. Therefore, the total residual deformation is calculated to be 5.325 mm.

#### Analysis of the stress

Upon completion of each load stage, the strain data for each measuring point was collected. Subsequently, the stress values for each measuring point were calculated and are presented in Table 2. Notably, when the load reached its maximum value of 5000 kN, the maximum stress recorded was 235.7 MPa, observed at the joint core area. This finding underscores the joint core area as the most critical location. In contrast, the minimum stress among the measuring points was 121.4 MPa, situated at the upper part of the main pipe.

As the load increased, the stress at each measuring point exhibited a linear increase during the initial four loading stages. However, upon reaching a load of 4750 kN, the stresses at measuring points situated within the joint core area stabilized at approximately 235 MPa. This phenomenon indicates the onset of a plastic zone within the joint core area. Additionally, when examining the measuring points arranged symmetrically along the circumference of the joint, it is evident that the stress levels at these points were largely consistent.

#### Discussion on limitations of the experiment

Limitations of load types: As this experiment only applied vertical loads, the experimental results may only be applicable to this specific type of load situation. In practical engineering, joints may be subjected to various different directions and types of loads.

Limitations of material properties: ZG20SiMn cast steel, as a test material, has specific mechanical properties and chemical composition. However, other types of cast steel or different materials may lead to different experimental results.

Neglecting time factors: Experiments are usually conducted within a limited time range and may not consider the long-term performance and durability of materials. For example, materials may undergo changes or aging over time, affecting their mechanical properties and structural stability.

In addition to these, there are limitations that can affect the results, such as environmental conditions, loading processes, boundary conditions, and human operations.

## Numerical simulation analysis

### Analysis model

**Figure 7:**
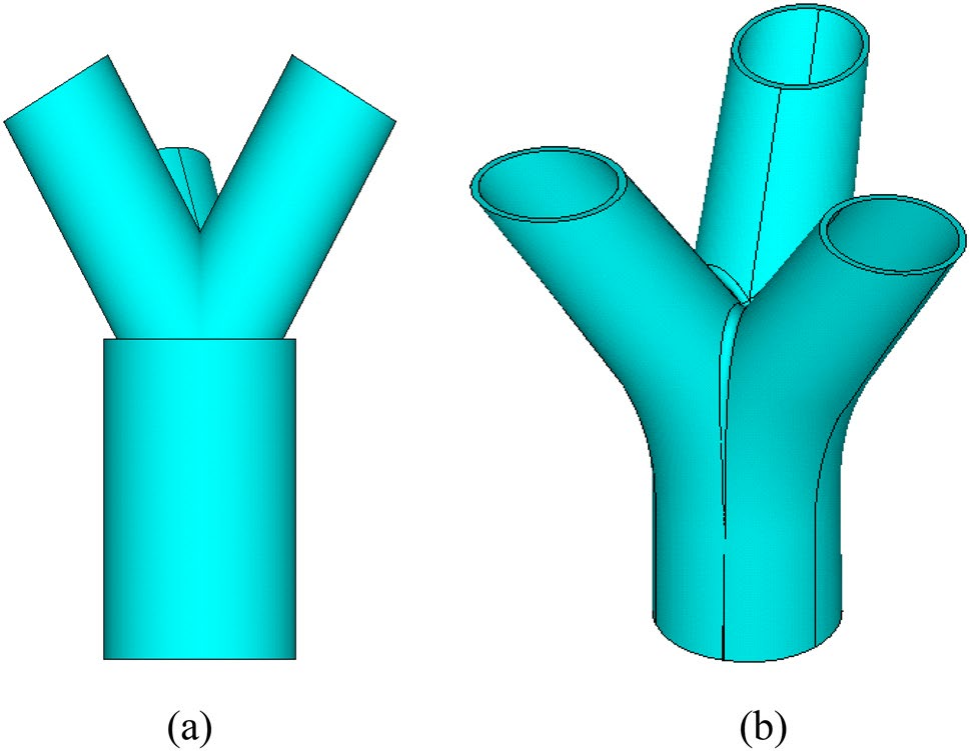
The comparison of joint model: (**a**) The model established by ANSYS; (**b**) The model established by SolidWorks.

To comprehensively assess the performance of the test joint, a corresponding finite element model with the same shape and size as the full-scale test was constructed. Initially, as depicted in Fig. 7a, the joint model was established using the default ANSYS preprocessor. However, challenges arose in achieving a seamless transition between the main pipe and the branches. Consequently, the joint was accurately modeled using SolidWorks version 2021(64-bit) software to ensure consistency with the actual joint configuration^23^, as illustrated in Fig. 7b.

**Figure 8:**
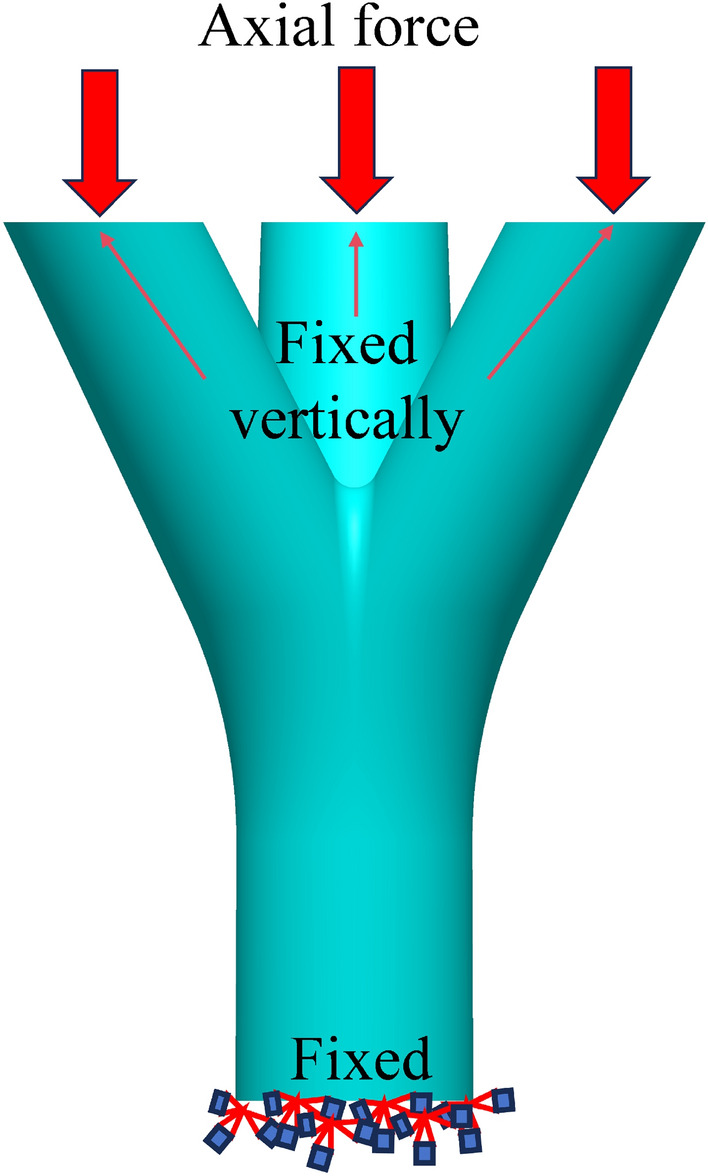
Boundary conditions.

The established joint model was imported into the finite element software ANSYS^24^ for analysis. The material properties used in the model were obtained from the material test. The elastic modulus of the material (*E*) was determined to be 2.0 × 10^5^ N/mm^2^, the yield strength (*f*_*y*_) was 235 MPa, and the Poisson's ratio (*μ*) was 0.3. The constitutive behavior of the material was selected to be the ideal elastic–plastic model, utilizing the Von-Mises yield criterion and associated flow rule for the elastic–plastic analysis^19^. To accurately simulate the real-world scenario, the boundary conditions of the joint were set as follows: the end part of the main pipe was fixed, while the ends of the branch pipes were fixed vertically. Additionally, the load was applied to the ends of the branch pipes in the form of surface pressure, as shown in Fig. 8.

**Figure 9:**
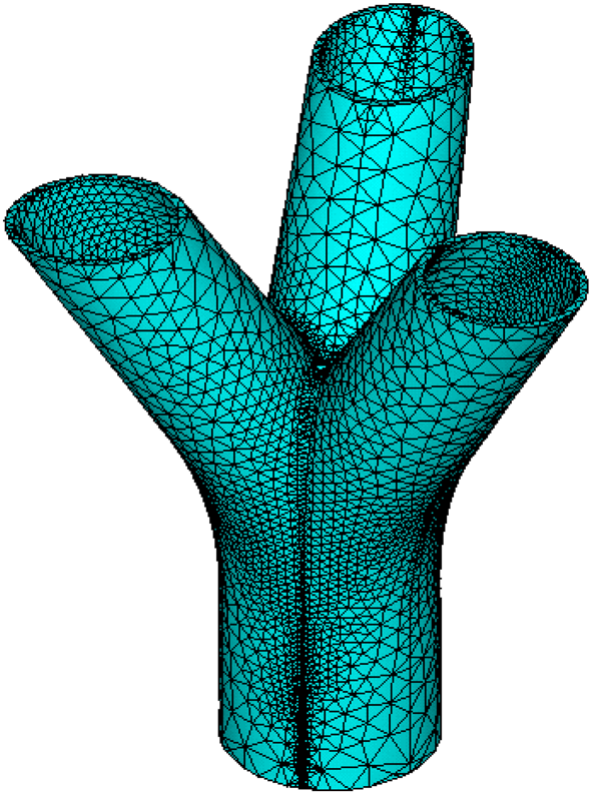
Finite element meshes.

After extensive research and comparison, the three-dimensional solid element Solid65 was selected from the ANSYS element type library for modeling the joint. This element type features quadratic displacement and is well-suited for irregular grid division^20^. We have previously conducted calculations and discussions on different finite element meshing schemes. It was found that the meshing near the branch junctions and the junctions between branches and the main pipe needs to be refined, while subdividing the wall thickness direction into several layers has minimal impact. When mesh elements are densely packed, the computational load increases, leading to longer computation times. However, overly dense meshing has minimal impact on the results. Therefore, after several attempts, we have settled on this meshing scheme. We adopted a free meshing approach with high-level meshing accuracy and localized mesh refinement. In the end, the nodes were divided into 61,988 elements. The finite element mesh of the joint, depicted in Fig. 9, was meticulously crafted to ensure accurate representation and analysis of the structural behavior.

### Analysis results of the testing joint

**Figure 10:**
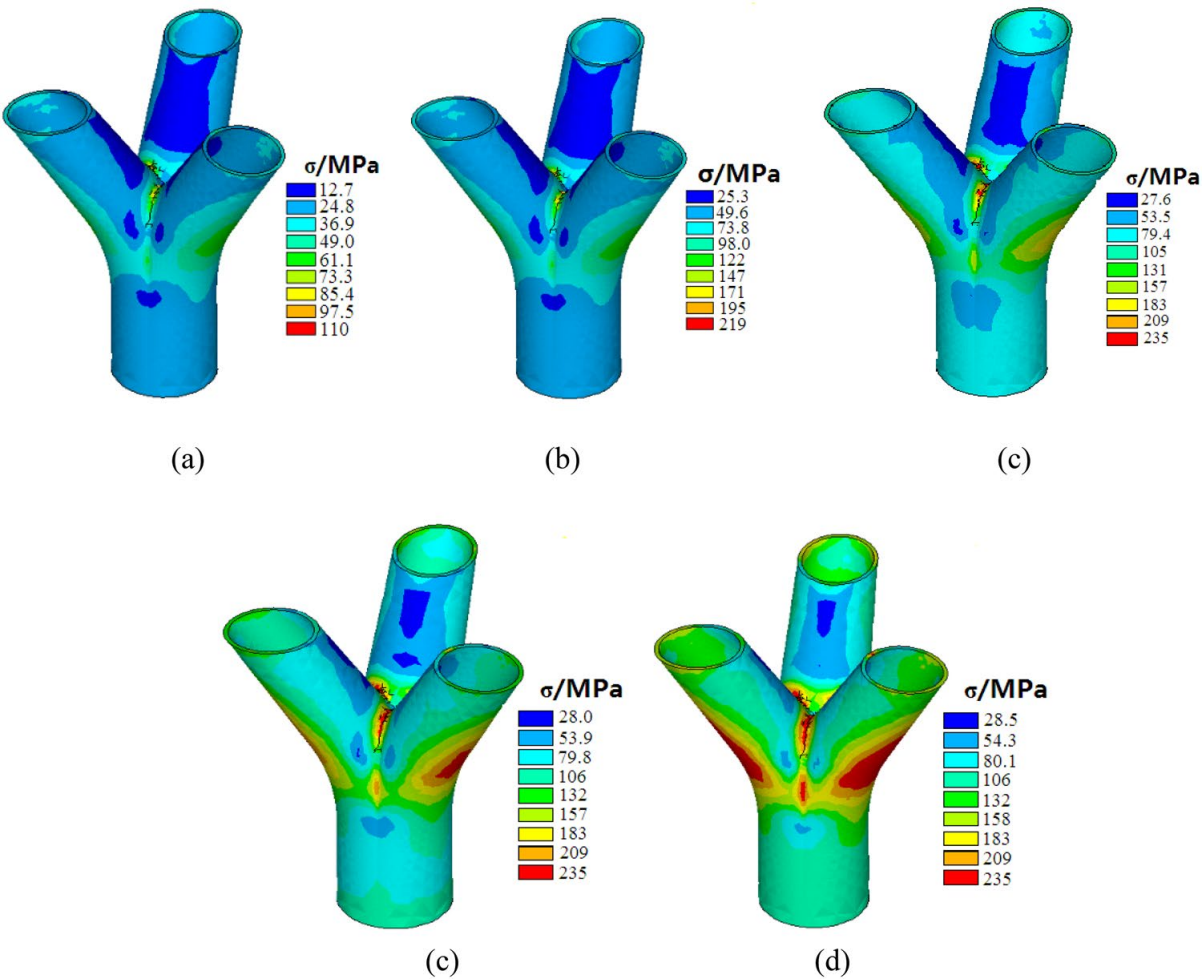
Stress contours of the joint: (**a**) The stress contour of the joint under 1000 kN; (**b**) The stress contour of the joint under 2000 kN; (**c**) The stress contour of the joint under 3000 kN; (**d**) The stress contour of the joint under 4000 kN; (**e**) The stress contour of the joint under 5000 kN.

The finite element analysis was conducted for the test joint under varying load conditions. Figure 10 illustrates the stress contours of the joint corresponding to load levels of 1000 kN, 2000 kN, 3000 kN, 4000 kN, and 5000 kN, respectively. From Fig. 10, it is evident that the overall stress level of the joint under a 1000 kN load is relatively low. The maximum stress value is predominantly concentrated in the vicinity of the joint core area. Furthermore, the stress observed in the main pipe and branch pipes is notably lower compared to that in the core area of the joint. Specifically, the stress in the former accounts for only 11.5% of the stress observed in the latter.

As the load increases to 2000 kN, the stress level of the joint exhibits a gradual linear increase, reaching a maximum stress value of 219 MPa. Upon reaching a load of 3000 kN, signs of yielding in the steel become apparent. However, the yield region is primarily concentrated at three points in the chamfer between the branch pipes. Subsequently, as the load further increases to 4000 kN, the yield region expands outward. This expansion manifests in two ways: firstly, the plastic region enlarges, and secondly, the chamfer between the main pipe and branch pipes also enters the plastic zone. Upon reaching the maximum load of 5000 kN, the expansion of the plastic zone within the joint intensifies, although it remains primarily concentrated in the vicinity of the joint core area. At this stage, nearly the entire core area of the joint enters the yield state, indicating the formation of a plastic hinge and marking the entry of the load–displacement curve into stage III (as illustrated in Fig. 6). Despite this, the stresses observed in the main pipe and branch pipes remain relatively low, approximately 106 MPa. This underscores the concentration of stress within the core region of the cast-steel joint with branches, which significantly influences its ultimate load-carrying capacity.

**Figure 11:**
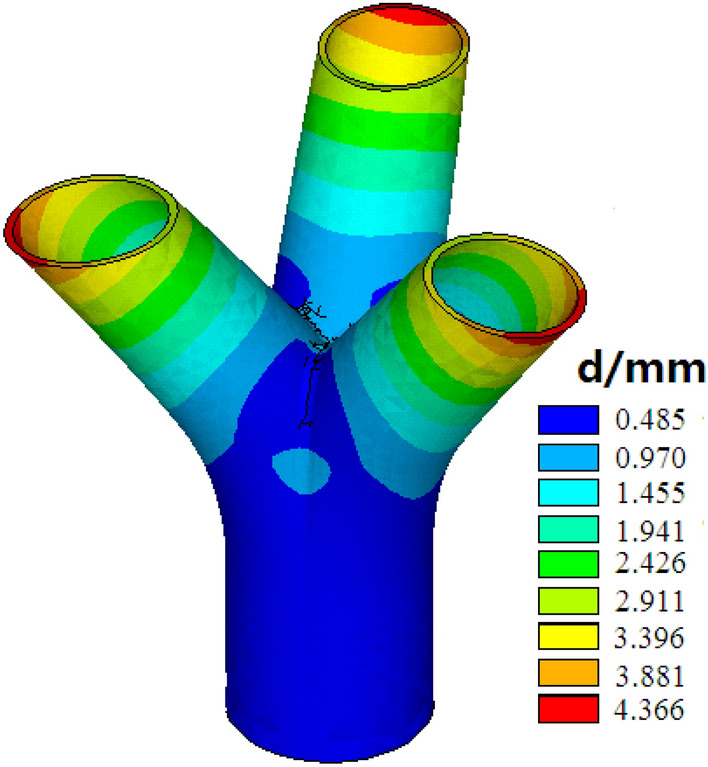
The vertical displacement contours of joint.

Figure 11 illustrates the vertical displacement of the joint under the maximum load of 5000 kN. It is observed that the maximum vertical displacement obtained from the finite element analysis (4.366 mm) is smaller than that obtained from the test (8.064 mm). This disparity can be primarily attributed to the non-tight contact between the joint and the test equipment piston during the experimental testing process.

### Verification of the finite element model through the experiment results

To validate the numerical model of the cast-steel joint with branches, a comparison is made between the results of finite element analysis and those of the verification experiment. The stress values obtained from representative measuring points in the finite element model are compared with the experimentally derived stress values, and their relative differences are listed in Table 3.

**Table 3:** Comparison between finite element analysis and experimental results.

Stress/MPa		Load/kN				
		1000	2000	3000	4000	5000
A2	Calculation results	24.8	49.6	79.4	106.8	132.2
	Experimental results	23.2	51.3	78.6	109.3	126.3
	Error analysis	6.89%	3.42%	1.01%	2.34%	4.67%
B12	Calculation results	33.4	71.5	105.3	139.3	169.8
	Experimental results	31.6	69.3	114.2	148.6	178.7
	Error analysis	5.69%	3.17%	8.45%	6.67%	5.24%
B22	Calculation results	36.9	73.8	112.5	157.2	182.1
	Experimental results	34.6	70.8	117.3	151.5	179.4
	Error analysis	6.64%	4.23%	4.27%	3.76%	1.50%
C12	Calculation results	48.3	90.2	135.4	183.2	209.4
	Experimental results	44.3	87.6	130.1	179.5	221.3
	Error analysis	9.02%	2.97%	4.07%	2.06%	5.68%
C22	Calculation results	49.1	91.5	130.2	180.2	235.0
	Experimental results	45.3	86.3	128.4	185.1	224.3
	Error analysis	8.39%	6.03%	1.40%	2.72%	4.77%
C32	Calculation results	48.7	89.9	138.6	179.6	230.2
	Experimental results	46.3	84.9	131.2	185.3	219.3
	Error analysis	5.18%	5.89%	5.64%	3.17%	4.97%

Analyzing the data in Table 3 reveals that:

1. The stress distribution of the cast-steel joint with branches obtained from the experiment aligns closely with that calculated from the finite element model. The calculated stresses from the finite element analysis and the experimental values of measuring points exhibit consistency. The maximum error between the calculated stresses and the experimental results is 9.02%, which occurs at the core area C12 under a vertical load of 1000kN. The calculated maximum stress is 48.3 MPa, while the experimental maximum stress is 44.3 MPa, substantiating the validity of the finite element model utilized in this study.
2. Both the finite element modeling and the experiment confirm that the area of large stress is concentrated in the core area of the joint. Under a vertical load of 5000kN, the maximum stress calculated in the core area C22 reaches 235 MPa, while the test also shows 224.3 MPa. The stresses in the main pipe and the branch pipes are comparatively small, and the maximum stress calculated for the main pipeline A2 is only 132.2 MPa, which is about half of the largest stress observed in the core area of the joint. Indicating that the failure of the three-way branching tree joint should be caused by the failure and fracture of the core area.
3. The casting precision of cast steel joints presents challenges in control. In this study, it was observed that the chamfer between the main pipe and the branch pipes was slightly larger than the design value. Additionally, the wall thickness exceeded the design specifications, resulting in a smaller diameter thickness ratio. Consequently, the stresses predicted by the finite element model tend to be generally higher than those observed in the experiment.
4. In summary, the results obtained from the finite element model align closely with those from the experiment. The numerical model effectively captures the actual stress and deformation states of the cast-steel joint with branches. Therefore, it can serve as a reliable tool for investigating the load-carrying capacity of such joints in further research.

### Effect of joint parameters on compression behavior of the cast-steel joint

To investigate the influence of different parameters on the ultimate load-carrying capacity of the joint, a parametric study is conducted wherein individual variables are varied while keeping other parameters constant. This approach allows for a systematic analysis of how each variable impacts the joint's performance independently.

The modeling results are shown in Table 4, which could be summarized as:

**Table 4:** The joints analysis results with different parameters under axial loading.

Joint number	*θ* (°)	*L* (mm)	*Β*	*γ*	*R*_1_ (mm)	*R*_2_ (mm)	*R*_3_ (mm)	Ultimate load-carrying capacity (kN)
J1	20	800	0.7	20	1000	0	0	2000.24
J2	30	800	0.7	20	1000	0	0	2301.79
J3	40	800	0.7	20	1000	0	0	2317.40
J4	50	800	0.7	20	1000	0	0	1660.00
J5	30	800	0.7	10	1000	0	0	5720.70
J6	30	800	0.7	15.2	1000	0	0	3344.50
J7	30	800	0.7	25	1000	0	0	1705.06
J8	30	800	0.7	29.9	1000	0	0	1334.73
J9	30	800	0.7	20	500	0	0	2056.27
J10	30	800	0.7	20	1500	0	0	2344.98
J11	30	800	0.7	20	2000	0	0	2233.56
J12	30	800	0.7	20	1000	10	0	2298.95
J13	30	800	0.7	20	1000	20	0	2268.51
J14	30	800	0.7	20	1000	30	0	2208.79
J15	30	800	0.7	20	1000	0	50	2824.84
J16	30	800	0.7	20	1000	0	100	3275.00
J17	30	800	0.7	20	1000	0	150	3296.32
J18	30	800	0.6	20	1000	0	0	2223.88
J19	30	800	0.66	20	1000	0	0	2281.90
J20	30	800	0.74	20	1000	0	0	2352.50
J21	30	800	0.8	20	1000	0	0	2418.69
J22	30	500	0.7	20	1000	0	0	2310.59
J23	30	600	0.7	20	1000	0	0	2312.61
J24	30	700	0.7	20	1000	0	0	2314.97

1. During compression testing, the fifth joint (J5) demonstrates the highest load-bearing capacity, reaching 5720.7 kN. Conversely, the eighth joint (J8) exhibits the lowest load-bearing capacity, registering only 1334.73 kN. Although both J8 and J5 share similar geometric characteristics, J8 boasts the greatest diameter thickness ratio (*γ*), while J5 possesses the smallest. Thus, the diameter thickness ratio (*γ*) significantly impacts the load-bearing capacity of joints.
2. Increasing only* θ* while holding other variables constant substantially decreases the joint's ultimate load-bearing capacity. This observation underscores *θ's* substantial influence on the joint's load-carrying capability.
3. Gradual increments in *β* and *R*_3_, while keeping other factors constant, substantially enhance the joint's ultimate load-carrying capacity. This finding highlights the significant impact of β and *R*_3_ on the joint's load-bearing capability.
4. When dimensions *L*, *R*_1_, and *R*_2_ undergo gradual increments while all other variables remain constant, the ultimate load-carrying capacity of the joint exhibits minimal variation, with the largest observed change being less than 5%. This indicates that dimensions *L*, *R*_1_, and *R*_2_ exert negligible influence on the ultimate load-carrying capacity of the joint.
5. Based on the findings of finite element modeling, it is deduced that thorough consideration of geometric parameters is imperative when analyzing the load-carrying capacity of cast-steel joints with branches. Careful selection of dimensional parameters for the joint is essential to ensure the structural safety and reliability.

## Load-carrying capacity estimation of the three-branch cast-steel joint

In existing literature^25,26^, load-carrying capacity formulas for welded tubular T-joints, steel tubular XK-joints, and multi-planar KX and KT-joints under axial loads are consistently represented as the product of the material yield strength and the square of the pipe wall thickness. Accordingly, the estimation of load-carrying capacity for cast-steel joints with branches can be similarly expressed as:

1 $$ F_{u} = KT^{2} f_{y} $$

where $F_{u}$ is load-carrying capacity of the joint; *K* is a parameter that contains the geometric parameters such as *θ*,* γ* and *β* of the joint; *T* is the pipe wall thickness; and *f*_*y*_ is the material yield strength of the joint.

In Eq. (1), the expression of parameter *K* serves as the primary research focus across various types of joints. Given that *K*encompasses a range of geometric parameters affecting the load-carrying capacity of the joint, the focus has shifted from solely examining the relationship between *K* and material yield strength (*F*_*u*_) to conducting multiple studies on the correlation between each individual parameter and *K*.

**Figure 12:**
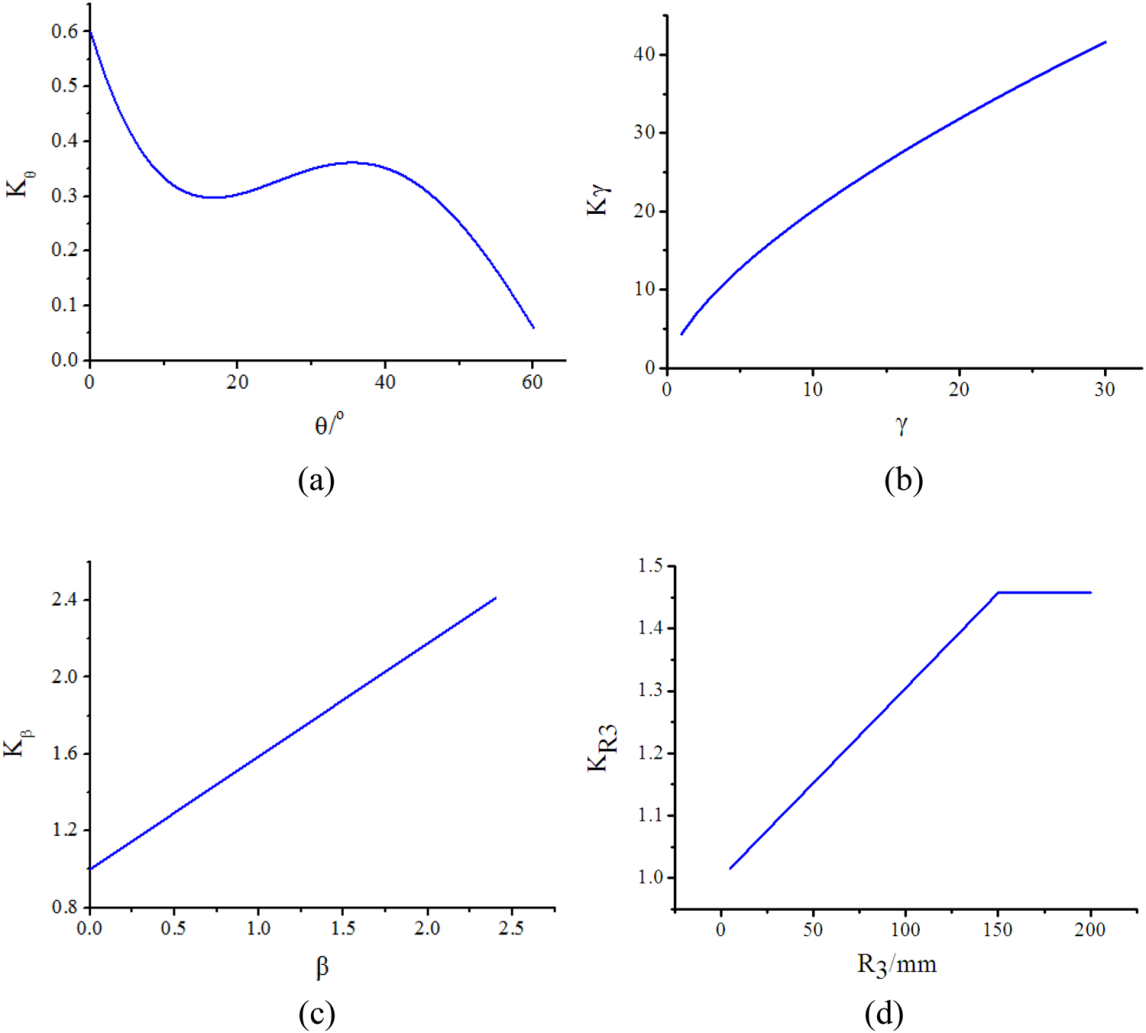
The relationship line chart between geometric parameters (*θ*, *γ*, *β*,* R*_3_) of the joint and the *K*: (**a**) Relationship line chart between *θ* and *K*_*θ*_; (**b**) Relationship line chart between *γ* and *Kγ*; (**c**) Relationship line chart between* β* and *K*_*β*_; (**d**) Relationship line chart between *R*_3_ and *K*_*R*3_.

The finite element analysis results indicate that dimensions *L*, *R*_1_, and *R*_2_ of the joint exert minimal influence on the ultimate load-carrying capacity. Consequently, these parameters are disregarded during the analysis of the comprehensive index *K*. Utilizing line charts depicting the relationships between *θ*, *γ*, *β*, and *R*_3_ with *K*, as illustrated in Fig. 12, a regression analysis is performed.

Following the regression analysis, the relationship between θ and Kθ is initially examined. Through this analysis, the relationship between the sine value of θ and Kθ can be expressed as:

2 $$ K_{\theta } = 0.60022 - 2.5311\sin \theta + 6.59681\sin^{2} \theta - 5.07388\sin^{3} \theta $$

For the relationship between γ and Kγ, it could be expressed as a power function:

3 $$ K_{\gamma } = 4.3725\gamma^{0.66242} $$

From the observations in Fig. 10, it is apparent that a linear relationship exists between β and Kβ. This relationship can be expressed as:

4 $$ K_{\beta } = 1 + 0.58856\beta $$

Finally, the regression analysis is performed between R3 and KR3. According to the principle of dimensional analysis, it is essential that the parameter R3 in the formula is dimensionless. To account for the influence of R3, a dimensionless chamfer coefficient ρ is defined as:

5 $$ \rho = \frac{{R_{3} }}{{\sqrt {dt} }} $$

where *d* is the outer diameter of the branch pipe; t is the wall thickness of the branch pipe. The finite element model shows that the joint ultimate load-carrying ca-pacity is very small when *R*_3_ is greater than or equal to 100 mm. So *R*_3_ is limited less than or equal to 100 mm on the ultimate load-carrying capacity calculation formula for the cast-steel joint with three branches. Through regression analysis, the relationship between the chamfer coefficient ρ and *K*_*R*3_ follows a linear relation, which is expressed:

6 $$ K_{{R_{3} }} = 1 + 0.33738\frac{{R_{3} }}{{\sqrt {dt} }} $$

Because these four parameters are independent of each other, the overall formula for the joint load-carrying capacity can be obtained by multiplying them, following the method of establishing the load-carrying capacity of joints in the existing standards, which is expressed as:

7 $$ \begin{gathered} F = 4.37251\gamma^{0.66242} (1 + 0.58856\beta )(1 + 0.33738\frac{{R_{3} }}{dt})(0.60022 - 2.5311\sin \theta + 6.59681\sin^{2} \theta - \hfill \\ 5.07388\sin^{3} \theta )f_{y} T^{2} \hfill \\ \end{gathered} $$

To validate the accuracy of Eq. (7), a comparison between the results obtained from finite element modeling and those derived from the regression formula is conducted. The comparative results are presented in Table 5. Notably, the disparity between the calculated values obtained from the formula and those from finite element analysis is minimal, with the maximum error amounting to only 1.9%. The formula proposed in this study exhibits good accuracy compared to the formulas obtained from references^27^ and^28^. The error in calculating the bearing capacity of K-shaped rectangular steel tube intersecting joints is within 20%, while the error in calculating the bearing capacity of Y-shaped cast steel joints is within 27.21%. Consequently, it is deduced that the proposed formula effectively predicts the ultimate load-carrying capacity of the cast-steel joint with three branches with a high level of accuracy. The derived formulas for bearing capacity in this study are obtained under the assumption that the main tube of the joint is fixed, while the other tubes are subjected to vertical axial loads. Therefore, these formulas are applicable only to cases where the joint is subjected to axial loads.

**Table 5:** Calculation formula error table.

Joint number	*θ* (°)	*γ*	*β*	*R*_3_ (mm)	*F/* (fyo*T^2^)	Regression formula results	Difference percentage (%)
J5	30	10	0.7	0	9.74	9.92	1.90
J6	30	15.2	0.7	0	13.07	13.09	0.19
J2	30	20	0.7	0	15.67	15.70	0.21
J7	30	25	0.7	0	18.14	18.21	0.37
J8	30	29.9	0.7	0	20.37	20.50	0.65
J1	20	20	0.7	0	13.62	13.62	0.00
J3	40	20	0.7	0	15.78	15.78	0.00
J4	50	20	0.7	0	11.30	11.30	0.00
J18	30	20	0.6	0	15.14	15.05	−0.61
J19	30	20	0.66	0	15.54	15.44	−0.61
J20	30	20	0.74	0	16.02	15.97	−0.32
J21	30	20	0.8	0	16.47	16.36	−0.66
J15	30	20	0.7	50	19.23	19.09	−0.75
J16	30	20	0.7	100	22.40	22.47	0.32

## Conclusion

This study investigates the bearing characteristics of triple-branched cast steel joints through full-scale tests, finite element analysis, and regression formula derivation. Initially, full-scale ultimate bearing capacity tests were conducted on triple-branched tree-like joints. Subsequently, ANSYS finite element analysis software was employed to simulate these joints under conditions considering geometric and material nonlinearities, as well as identical boundary conditions and loading modes as the experiments, to validate the accuracy of the finite element simulations. Furthermore, finite element analysis was used to analyze the bearing capacity of 24 triple-branched tree-like joints with different geometric parameters, and regression methods were employed to establish derivation formulas for the bearing capacity of triple-branched cast steel joints. Through the extensive research conducted in this paper, the following conclusions were drawn:

1. Analysis of the full-scale joint experimental results revealed that stress distribution under compression primarily concentrates on the core area of the joint, with minimal stress observed in the main pipe and branch pipes. This insight serves as a basis for evaluating the joint's strength and stiffness to meet design requirements.
2. The finite element model of the test joint was imported into ANSYS for analysis, and the results were compared with experimental findings, demonstrating consistency. The verified finite element model is deemed reliable for evaluating the impact of joint geometry parameters on the behavior of three-branch cast-steel joints.
3. Finite element analysis was conducted on joints with various geometric parameters to determine their ultimate load-carrying capacities. The error analysis revealed a maximum error of 1.9% when comparing prediction results with finite element results, indicating that the proposed formula accurately predicts ultimate load-carrying capacity for engineering design requirements. The proposed formulas fill the gap in estimating the bearing capacity of branched tree-like cast steel joints under axial loads, making them an essential tool for conducting research on joint bearing capacity and joint design.
4. The proposed derivation formulas for bearing capacity also enhance the efficiency and accuracy of joint design. In design, the obtained bearing capacity calculation formulas allow for the determination of corresponding dimensions for branches, main tubes, and the deviation angles between branches and the main tube under known bearing capacity conditions. This provides a theoretical foundation for future research and design of branched tree-like joints. Furthermore, future studies can explore the development of universal formulas for bearing capacity or stiffness of tree-like branch joints with different numbers of branches.

## Data Availability

The datasets used and analyzed during the current study are available from the corresponding author on reasonable request.
